# Anisotropic Gaussian kernel adaptive filtering by Lie-group dictionary learning

**DOI:** 10.1371/journal.pone.0237654

**Published:** 2020-08-14

**Authors:** Tomoya Wada, Kosuke Fukumori, Toshihisa Tanaka, Simone Fiori

**Affiliations:** 1 Department of Electrical and Electronic Engineering, Tokyo University of Agriculture and Technology, Koganei-shi, Tokyo, Japan; 2 Università Politecnica delle Marche, Ancona, Italy; Newcastle University, UNITED KINGDOM

## Abstract

The present paper proposes a novel kernel adaptive filtering algorithm, where each Gaussian kernel is parameterized by a center vector and a symmetric positive definite (SPD) precision matrix, which is regarded as a generalization of scalar width parameter. In fact, different from conventional kernel adaptive systems, the proposed filter is structured as a superposition of non-isotropic Gaussian kernels, whose non-isotropy makes the filter more flexible. The adaptation algorithm will search for optimal parameters in a wider parameter space. This generalization brings the need of special treatment of parameters that have a geometric structure. In fact, the main contribution of this paper is to establish update rules for precision matrices on the Lie group of SPD matrices in order to ensure their symmetry and positive-definiteness. The parameters of this filter are adapted on the basis of a least-squares criterion to minimize the filtering error, together with an ℓ_1_-type regularization criterion to avoid overfitting and to prevent the increase of dimensionality of the dictionary. Experimental results confirm the validity of the proposed method.

## 1 Introduction

Adaptive filtering is a technique to update the parameters of a signal/data processing structure [1]. In this paper, we deal with kernel-based adaptive filtering. A kernel adaptive filter is a kind of nonlinear filter that exploits a kernel method, which is a technique to construct effective nonlinear systems [2]. Kernel adaptive filters found widespread applications in diverse fields, ranging from stock market prediction [3] to acoustic echo cancellation [4] and visual object tracking [5].

In kernel adaptive filtering, most kernels present the following form [6]:

$$\begin{array}{c} \kappa \left(\cdot ,c;\gamma \right)≔\exp({-\gamma ∥\cdot -c∥}^{2}), \end{array}$$ (1)

where parameters $c\in R^{L}$ and *γ* > 0 represent the *center* and the *width* of a Gaussian kernel, respectively. In other words, this kind of kernel presents only two parameters, namely, mean and variance (also referred to as scalar precision).

### 1.1 Related work

Several instances of nonlinear adaptive filtering have been reported in the scientific literature. Among them, kernel adaptive filtering developed in a reproducing kernel Hilbert space (RKHS) is known as an efficient online nonlinear approximation approach [7, 8]. Well-known kernel adaptive filtering algorithms are kernel least mean square (KLMS) [9–12], kernel normalized least mean square (KNLMS), kernel affine projection algorithms (KAPA) [13, 14], and kernel recursive least squares (KRLS) [15]. In this context, it is worth citing fractional adaptive signal processing [16–20] as these modern filtering algorithms outperform their counterparts in terms of accuracy and convergence, for example in active noise control systems.

A distinguishing feature of kernel-based adaptive filtering is the ability to adjust the values of the parameters of each kernel so as to minimize the filtering error. To what concerns kernel centers, research endeavors suggested to move all the center vectors in the dictionary to minimize the squared filtering error [21–23]. To what concerns kernel widths, it is known that the widths of the kernels are important parameters that contribute to improve the performance of kernel machines [24–28] and some attempts to adaptively estimate the widths of the kernels have been reported [27, 28]. Moreover, in a recent work [6], Wada *et al.* have proposed an adaptive update method for both the Gaussian center and width concurrently. The above mentioned papers have addressed the problem of estimating a precision parameter of the Gaussian model given as in (1). However, this is a special case of multivariate Gaussian kernel function.

The structure of most kernel adaptive filtering algorithms grows linearly with each new input sample. A solution to cope with this problem is to build a *dictionary*. Well-known criteria for dictionary learning are novelty [29], approximate linear dependency (ALD) [15], surprise [30], and coherence-based criterion [31]. Another known criterion is ℓ_1_-regularization [32, 33], which sets some coefficients to zero and discards the corresponding entries. In this instance, a model dynamically changes, in that new members may be added to a dictionary and old members may be suppressed from a dictionary.

### 1.2 Innovative contribution

It should be noted that a kernel of the form (1) implicitly assumes uncorrelatedness between components in the sample vector, which implies that the kernel can be isotropic. However, observed samples usually present some sort of mutual correlation [34, 35].

In this paper, we employ a generalized Gaussian kernel defined as

$$\begin{array}{c} \kappa \left(\cdot ,c;\Gamma \right)≔\exp(-{\left(\cdot -c\right)}^{⊤}\Gamma \left(\cdot -c\right)), \end{array}$$ (2)

where $\Gamma \in R^{L\times L}$ is a symmetric positive definite (SPD) matrix. We refer to Γ as a precision matrix, which has no constraint but positive definiteness, while the model of (1) can be regarded as a special case where Γ = *γ**I***. In other words, in (1) the precision matrix is allowed to be only isotropic. Unlike (1), this general form has more degrees of freedom and therefore it is more flexible in modeling samples distributions; however, an adaptive method for finding the precision matrix is not straightforward. We will establish a dictionary learning method for generalized Gaussian kernel adaptive filtering. In a dictionary, each entry consists of a pair formed by a center vector and a precision matrix. The main contributions of the proposed method are (a) a model of the filter consisting of kernels with a different precision matrix each (b) an update rule for each center vector, and (c) a learning rule for each precision matrix on the SPD manifold in order to ensure their symmetry and positivity definiteness during adaptation.

### 1.3 Organization and list of abbreviations

Section 2 presents general concepts in kernel adaptive filtering. Section 3 proposes a dictionary learning method for the generalized Gaussian kernel adaptive filtering. Section 4 shows the results of numerical experiments to evaluate the efficacy of the proposed method. Section 5 concludes the paper. A list of abbreviations used within this paper is presented in Table 1.

**Table 1:** List of abbreviations and their meaning.

Abbreviation	Explanation
ALD	approximate linear dependency
KAPA	kernel affine projection algorithms
KLMS	kernel least mean square
KNLMS	kernel normalized least mean square
KRLS	kernel recursive least squares
LMS	least mean squares
MEG	matrix exponentiated gradient
MSE	mean squared error
NMEG	normalized matrix exponentiated gradient
RKHS	reproducing kernel Hilbert space
SPD	symmetric positive definite

## 2 Kernel adaptive filters

Kernel adaptive filters possess noteworthy features [8], such as universal approximation ability, absence of local minima and moderate complexity in terms of computation burden and memory. In this section, we first discuss sample distributions modeling in the context of kernel adaptive filtering and then we briefly review kernel adaptation algorithms.

**Fig 1:**
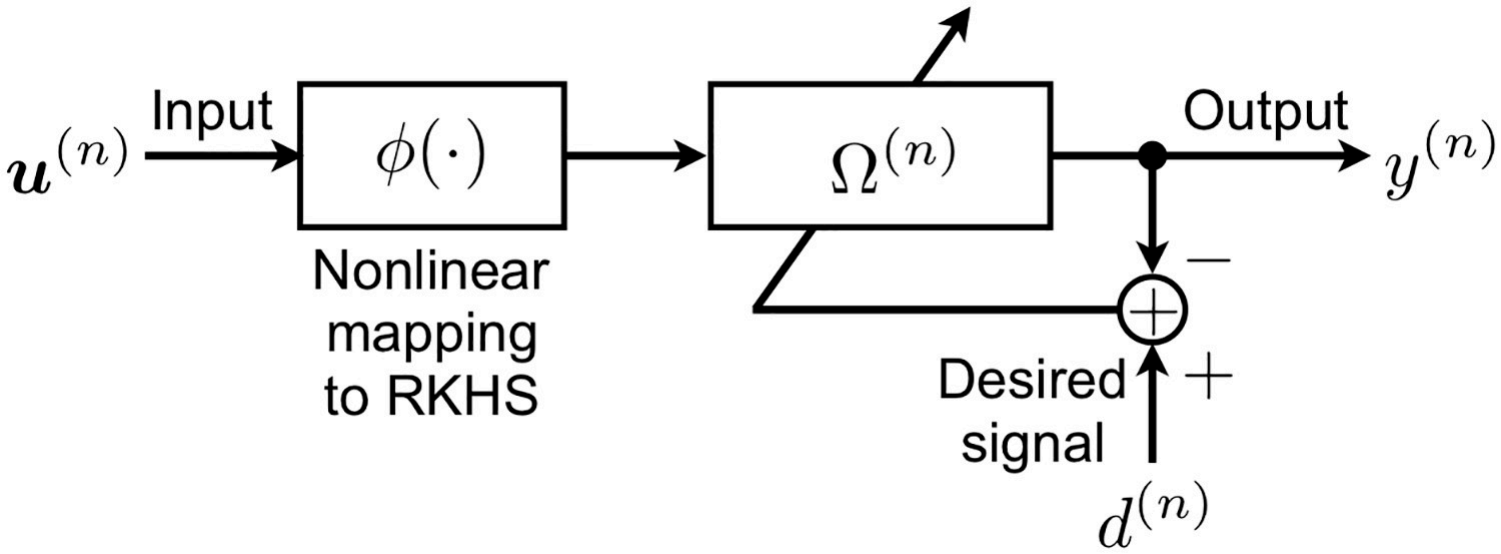
Schematic of a RKHS adaptive filter.

In kernel adaptive filtering, an input sequence $u^{\left(n\right)}\in U\subset R^{L}$ is mapped to a RKHS (H,〈·,·〉) on U induced from a positive definite kernel κ(·,·):U×U→R. Here, symbol U denotes a multidimensional input space, while symbol 〈·,·〉:H×H→R denotes an inner product in the RKHS. A RKHS H can implicitly increase the dimensionality of a feature space that enables us to represent non-linear signals, which are generated by a non-linear system [36]. The short-term scalar output sequence of the filter is computed as

$$\begin{array}{c} y^{\left(n\right)}=\left\langle \varphi \left(u^{\left(n\right)}\right),P^{\left(n\right)}\right\rangle , \end{array}$$ (3)

where $P^{\left(n\right)}\in H$ denotes a filter weight vector at time *n* and ϕ:U→H denotes a nonlinear mapping. In general, the inner product in a high dimensional space is not given in an explicit form. Rather, the inner product in a RKHS can be calculated by using the properties of RKHS, namely: (i) all elements in a RKHS are constructed by a kernel *κ*(⋅, ⋅), (ii) it is convenient to choose *φ*(***u***) = *κ*(⋅, ***u***), (iii) it holds that 〈*κ*(⋅, ***u***_*i*_), *κ*(⋅, ***u***_*j*_)〉 = *κ*(***u***_*i*_, ***u***_*j*_) [2, 31]. The Fig 1 shows a schematic of the adaptive filter.

We consider the problem of adaptively estimating the weights *P*^(*n*)^. It is known [31] that *P*^(*n*)^ can be written as

$$\begin{array}{c} P^{\left(n\right)}=\sum_{j\in J^{\left(n\right)}}h_{j}^{\left(n\right)}\kappa \left(\cdot ,c_{j}\right), \end{array}$$ (4)

where the $h_{j}^{\left(n\right)}\in R$ are scalar weight coefficient for *κ*(⋅, ***c***_*j*_). Here, ${\left\{c_{j}\right\}}_{j\in J^{\left(n\right)}}$ is a set of input samples, termed *dictionary*. The symbol $J^{\left(n\right)}$ denotes an index set of dictionary elements at time *n*. From Eqs (3) and (4), the filter output is written as

$$\begin{array}{c} y^{\left(n\right)}=\sum_{j\in J^{\left(n\right)}}h_{j}^{\left(n\right)}\kappa \left(u^{\left(n\right)},c_{j}\right)={h^{\left(n\right)}}^{⊤}\kappa^{\left(n\right)}, \end{array}$$ (5)

where

$$\begin{array}{c} h^{\left(n\right)}≔{\left[h_{j_{1}^{\left(n\right)}}^{\left(n\right)},h_{j_{2}^{\left(n\right)}}^{\left(n\right)},\ldots \right]}^{⊤},\kappa^{\left(n\right)}≔{\left[\kappa \left(u^{\left(n\right)},c_{j_{1}^{\left(n\right)}}\right),\kappa \left(u^{\left(n\right)},c_{j_{2}^{\left(n\right)}}\right),\ldots \right]}^{⊤}. \end{array}$$ (6)

Notice that, in the above equations, for the sake of notation conciseness the kernel functions have been indicated without reference to the kernel width parameter *γ* that takes the same value in each kernel.

In kernel adaptive filtering algorithms, obsolete kernel functions cannot be discarded, which is a serious limitation of these algorithms, in particular in the presence of a non-stationary environment. In order to fix this issue, a dictionary may be constructed by means of ℓ_1_-regularization [32, 33] which promotes sparsity, hence improving the efficiency of the dictionary. Let $d^{\left(n\right)}\in R$ denotes a desired output signal at time *n*. The cost function for the adaptive kernel algorithm is written as follows:

$$\begin{array}{c} A^{\left(n\right)}≔{\left|d^{\left(n\right)}-{h^{\left(n\right)}}^{⊤}\kappa^{\left(n\right)}\right|}^{2}+\mu \underset{≕\beta^{\left(n\right)}}{\underset{︸}{\sum_{j\in J^{\left(n\right)}}w_{j}^{\left(n\right)}\left|h_{j}^{\left(n\right)}\right|}}, \end{array}$$ (7)

where *β*^(*n*)^ and *μ* play the role of a weighted ℓ_1_ norm and of a regularization parameter, respectively. Here, the weights ${\left\{w_{j}^{\left(n\right)}\right\}}_{j\in J^{\left(n\right)}}$ are dynamically adjusted as $w_{j}^{\left(n\right)}=1/\left(|h_{j}^{\left(n\right)}|+\rho \right)$ [33], with a small constant *ρ* > 0 to prevent the denominator from vanishing.

It is worth noticing that a conventional stochastic gradient descent method would be ineffective to seek the minimum of the cost function (7) since the weighted ℓ_1_ norm is not smooth. However, since the cost function *A*^(*n*)^ is convex, a forward-backward splitting scheme [37] may be applied. A forward-backward splitting scheme reads:

$$\begin{array}{c} h^{\left(n+1\right)}={\text{prox}}_{\lambda \mu \beta^{\left(n\right)}}[\bar{h^{\left(n\right)}}+\frac{\mu (d^{\left(n\right)}-{\bar{h^{\left(n\right)}}}^{⊤}\bar{\kappa^{\left(n\right)}})\bar{\kappa^{\left(n\right)}}}{\sigma +{∥\bar{\kappa^{\left(n\right)}}∥}^{2}}], \end{array}$$ (8)

where $\bar{h^{\left(n\right)}}≔{\left[{h^{\left(n\right)}}^{⊤},0\right]}^{⊤}$, $\bar{\kappa^{\left(n\right)}}≔{\left[{\kappa^{\left(n\right)}}^{⊤},\kappa \left(u^{\left(n\right)},u^{\left(n\right)}\right)\right]}^{⊤}$, the coefficient λ > 0 denotes a step size, the coefficient *σ* denotes a stabilization parameter, and ‖⋅‖ denotes a standard vector 2-norm. The symbol ‘prox’ denotes the proximal operator [37], which is defined as follows: given a vector $q≔{\left[q_{1},q_{2},\ldots ,q_{r}\right]}^{⊤}\in R^{r}$, it holds that

$$\begin{array}{c} {\left({\text{prox}}_{\lambda \mu \beta^{\left(n\right)}}\left(\alpha \right)\right)}_{j}≔\text{sgn}\left\{q_{j}\right\}\max\{|q_{j}\left|-\lambda \mu w_{j}^{\left(n\right)},0\right\}. \end{array}$$ (9)

For further details on this technique, interested readers might consult [6, 38, 39]. This learning rule promotes the sparsity of the $h_{j}^{\left(n\right)}$’s, which results in some coefficient $h_{j}^{\left(n\right)}$ approaching zero and the corresponding center vector **c**_*j*_ getting removed from the dictionary.

## 3 Model and dictionary learning for generalized Gaussian kernel adaptive filtering

As recalled in the introduction, most kernel machines using Gaussian kernel functions implicitly assume uncorrelatedness within the sample-variables, even though observed samples usually present correlation. In the following, a flexible filtering structure based on a superposition of generalized Gaussian functions is proposed and algorithms for learning its parameters are established.

### 3.1 Adaptive kernel filter based on a superposition of generalized Gaussian functions

The proposed model is structured as a superposition of generalized Gaussian kernels given as in (2) with time-varying centers $c_{j}^{\left(n\right)}$ and precision matrices $\Gamma_{j}^{\left(n\right)}$. The output sequence of one such kernel adaptive filter is computed as

$$\begin{array}{cc} y^{\left(n\right)} & =\sum_{j\in J^{\left(n\right)}}{h_{j}}^{\left(n\right)}\kappa \left(u^{\left(n\right)},c_{j}^{\left(n\right)};\Gamma_{j}^{\left(n\right)}\right)\\ & =\sum_{j\in J^{\left(n\right)}}{h_{j}}^{\left(n\right)}\exp(-{\left(u^{\left(n\right)}-c_{j}^{\left(n\right)}\right)}^{⊤}\Gamma_{j}^{\left(n\right)}\left(u^{\left(n\right)}-c_{j}^{\left(n\right)}\right)). \end{array}$$ (10)

The corresponding dictionary at time *n* is described by

$$\begin{array}{c} D^{\left(n\right)}≔\left\{\left(c_{j_{1}}^{\left(n\right)},\Gamma_{j_{1}}^{\left(n\right)}\right),\left(c_{j_{2}}^{\left(n\right)},\Gamma_{j_{2}}^{\left(n\right)}\right),\ldots ,\left(c_{j_{r^{\left(n\right)}}}^{\left(n\right)},\Gamma_{j_{r^{\left(n\right)}}}^{\left(n\right)}\right)\right\}. \end{array}$$ (11)

For the sake of completeness, let us discuss how a multikernel adaptive filter fits within the general theory of RKHS. An extended discussion on multikernel adaptive filtering may be found in [38, 40].

Let $H_{1}$ and $H_{2}$ denote two reproducing kernel Hilbert spaces and let $H≔H_{1}⊕H_{2}$ denote their direct sum. The norm of the direct sum of $f_{1}\in H_{1}$ and $f_{2}\in H_{2}$, *f* = (*f*_1_, *f*_2_)∈*H*, is represented as [2]:

$$\begin{array}{c} {∥f∥}_{H}^{2}≔∥f_{1}{∥}_{H_{1}}^{2}+{∥f_{2}∥}_{H_{2}}^{2}. \end{array}$$ (12)

In particular, if the two Hilber spaces are non-overlapping, namely $H_{1}\cap H_{2}=\left\{0\right\}$, the sum space $H≔\{f=f_{1}+f_{2}|f_{1}\in H_{1},f_{2}\in H_{2}\}$ has the same structure of the space *H* [2]. Consequently, the norm in H may be defined as:

$$\begin{array}{c} {∥f∥}_{H}^{2}≔∥f_{1}{∥}_{H_{1}}^{2}+{∥f_{2}∥}_{H_{2}}^{2}. \end{array}$$ (13)

Also, take a kernel $\kappa_{1}\in H_{1}$ and a kernel $\kappa_{2}\in H_{2}$. An element f∈H can be evaluated by the sum kernel *κ* ≔ *κ*_1_+ *κ*_2_ [2]:

$$\begin{array}{c} f\left(u\right)={\left\langle f,\kappa (\cdot ,u)\right\rangle }_{H}={\left\langle f_{1},\kappa_{1}\left(\cdot ,u\right)\right\rangle }_{H_{1}}+{\left\langle f_{2},\kappa_{2}\left(\cdot ,u\right)\right\rangle }_{H_{2}}. \end{array}$$ (14)

The above construction may be generalized to an arbitrary number of Hilbert spaces without difficulty.

Assume now that *M* different kernels ${\left\{\kappa_{m}\left(\cdot ,\cdot \right)\right\}}_{m=1}^{M}$ are available. Denote by $H_{m}$ a RKHS determined by the *m*-th kernel and define H as the corresponding sum space. In analogy to the simpler case (14), the output of the filter is obtained by combining a weight P∈H and the ‘sum kernel’ κ∈H as

$$\begin{array}{c} y^{\left(n\right)}={\left\langle P,\kappa \left(\cdot ,u^{\left(n\right)}\right)\right\rangle }_{H}=\sum_{m=1}^{M}{\left\langle P_{m},\kappa_{m}\left(\cdot ,u^{\left(n\right)}\right)\right\rangle }_{H_{m}}, \end{array}$$ (15)

where each weight $P_{m}\in H_{m}$ and *P* is identified with the (direct) sum of the single weights *P*_*m*_. Since there is no need for the index set of the dictionary in each RKHS to equate each other [38], the filter structure (10) may be identified as a multikernel adaptive filter with time-varying weights:

$$\begin{array}{c} y^{\left(n\right)}={\left\langle P^{\left(n\right)},\kappa \left(\cdot ,u^{\left(n\right)}\right)\right\rangle }_{H}=\sum_{j\in J^{\left(n\right)}}{\left\langle P_{j}^{\left(n\right)},\kappa \left(\cdot ,u^{\left(n\right)};\Gamma_{j}^{\left(n\right)}\right)\right\rangle }_{H_{j}}, \end{array}$$ (16)

with the convention that $P_{j}^{\left(n\right)}≔h_{j}^{\left(n\right)}\kappa \left(\cdot ,c_{j}^{\left(n\right)};\Gamma_{j}^{\left(n\right)}\right)$.

### 3.2 Center vectors adaptation

In this subsection, a dictionary learning method for generalized Gaussian kernel adaptive filtering is proposed. To update the center vectors, we chose the loss function:

$$\begin{array}{cc} F^{\left(n\right)}\left(D^{\left(n\right)}\right) & ≔|e^{\left(n\right)}{|}^{2}={\left|d^{\left(n\right)}-y^{\left(n\right)}\right|}^{2}\\ & ={\left|d^{\left(n\right)}-\sum_{j\in J^{\left(n\right)}}h_{j}^{\left(n\right)}\exp(-{\left(u^{\left(n\right)}-c_{j}\right)}^{⊤}\Gamma_{j}^{\left(n\right)}\left(u^{\left(n\right)}-c_{j}\right))\right|}^{2}. \end{array}$$ (17)

Such criterion is a function of dictionary elements, namely, of center vectors as well as of precision matrices.

The adaptation of each center vector may be achieved by a gradient steepest descent algorithm:

$$\begin{array}{c} c_{j}^{\left(n+1\right)}=c_{j}^{\left(n\right)}-\eta_{\text{c}}\frac{\partial F^{\left(n\right)}(c_{j})}{\partial c_{j}}{|}_{c_{j}=c_{j}^{\left(n\right)}} \end{array}$$ (18)

where *η_c_* > 0 denotes a step size and

$$\begin{array}{c} \frac{\partial F^{\left(n\right)}(c_{j})}{\partial c_{j}}|c_{j}=c_{j}^{\left(n\right)}=-4e^{\left(n\right)}h_{j}^{\left(n\right)}\kappa (u^{\left(n\right)},c_{j}^{\left(n\right)};\Gamma_{j}^{\left(n\right)})\Gamma_{j}^{\left(n\right)}(u^{\left(n\right)}-c_{j}^{\left(n\right)}) \end{array}$$ (19)

Let us remark that adaptation rules to move center vectors for the standard Gaussian kernel adaptive filters were also proposed in [21–23].

### 3.3 Precision matrices adaptation

**Fig 2:**
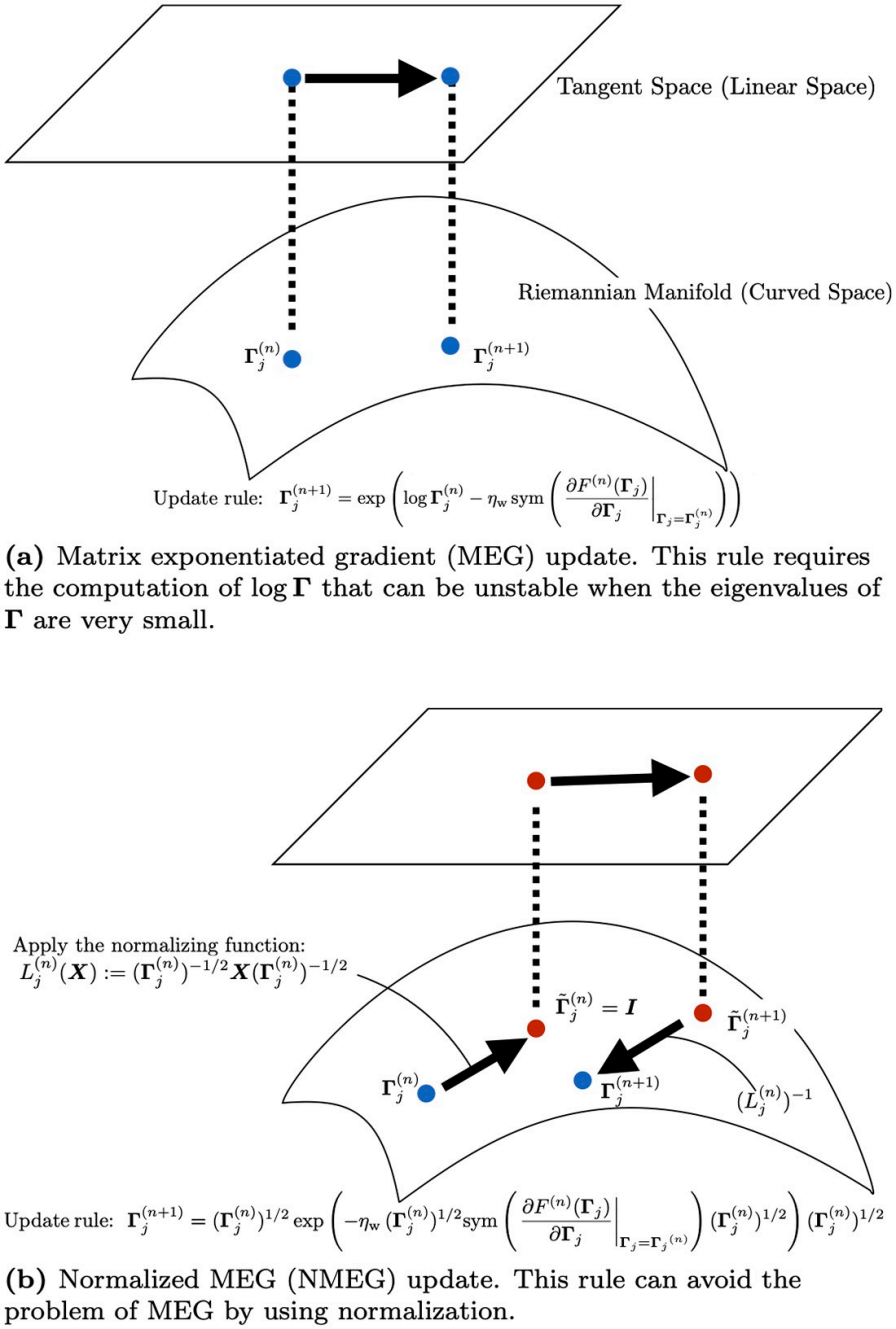
Conceptual diagrams of (a) MEG and (b) NMEG.

In order to update the precision matrices, we consider two types of data-driven adaptation methods. One consists in applying the update rule for SPD matrices proposed in [41]. The other is a novel update rule where an effective normalization is employed. The Fig 2 illustrates, in a schematic way, these update rules. In order to update precision matrices, the same loss function (17) may be invoked.

#### 3.3.1 Matrix Exponentiated Gradient (MEG) adaptation

To update the precision matrices in a dictionary ${\left\{\Gamma_{j}\right\}}_{j\in J^{\left(n\right)}}$ while preserving their SPD structure, a matrix exponentiated gradient (MEG) update [41] may be applied. The update rule for Γ_*j*_ can be derived to minimize the loss function in (17):

$$\begin{array}{c} \Gamma_{j}^{\left(n+1\right)}=\text{exp}(\text{log}\Gamma_{j}^{\left(n\right)}-\eta_{\text{w}}\;\text{sym}(\frac{\partial F^{\left(n\right)}(\Gamma_{j})}{\partial \Gamma_{j}}{|}_{\Gamma_{j}=\Gamma_{j}^{\left(n\right)}})), \end{array}$$ (20)

where *η*_w_ > 0 denotes a step size and

$$\begin{array}{c} \frac{\partial F^{\left(n\right)}(\Gamma_{j})}{\partial \Gamma_{j}}{|}_{\Gamma_{j}=\Gamma_{j}^{\left(n\right)}}=\Gamma_{j}^{\left(n\right)}=2e^{\left(n\right)}h_{j}^{\left(n\right)}\kappa \left(u^{\left(n\right)},c_{j}^{\left(n\right)};\Gamma_{j}^{\left(n\right)}\right)\left(u^{\left(n\right)}-c_{j}\right){\left(u^{\left(n\right)}-c_{j}\right)}^{⊤}. \end{array}$$

For a square matrix **X**, $\text{sym}\left(X\right)≔\frac{1}{2}\left(X+X^{⊤}\right)$ denotes the symmetric part of **X**, while exp(**X**) and log(**X**) denote matrix exponential and principal matrix logarithm, respectively [41].

It is interesting to observe that the adaptation rule (20) may be re-interpreted in the framework of manifold calculus. In fact, let us define a retraction [42] as *R*_**X**_(**V**) ≔ exp(log**X** + **V**) in the SPD space, where **X** denotes any positive-definite symmetric matrix and **V** denotes any symmetric matrix of the same size. Also, let us define $\nabla_{X}F≔\text{sym}(\frac{\partial F}{\partial X})$ as the Riemannian gradient of a loss function *F* with respect to the SPD matrix **X** [43]. Then, the adaptation rule (20) may be re-framed as $\Gamma_{j}^{\left(n+1\right)}=R_{\Gamma_{j}^{\left(n\right)}}(-\eta_{w}\nabla_{\Gamma_{j}^{\left(n\right)}}F^{\left(n\right)})$.

#### 3.3.2 Normalized Matrix Exponentiated Gradient (NMEG) adaptation

Even though matrix exponentiated gradient updates each precision matrix Γ while preserving its SPD structure, the computation of logΓ can be unstable when the eigenvalues of Γ are too close to zero. A symmetric positive-definite matrix Γ with *L* all-distinct eigenvalues may be decomposed as ***W*** *diag*(λ_1_, λ_2_, …, λ_*L*_)***W***^⊤^, with **W** orthogonal. Therefore, logΓ = ***W*** *diag*(logλ_1_, logλ_2_, …, logλ_*L*_)**W**^⊤^: If an eigenvalue lays too close to zero, matrix logarithm becomes numerically unstable. In general, a matrix logarithm is well-defined only in a neighbor of the identity matrix **I**. To overcome this problem, the following normalizing function by the current value $\Gamma_{j}^{\left(n\right)}$ is proposed:

$$\begin{array}{c} L_{j}^{\left(n\right)}\left(X\right)≔{\left(\Gamma_{j}^{\left(n\right)}\right)}^{-1/2}X{\left(\Gamma_{j}^{\left(n\right)}\right)}^{-1/2}, \end{array}$$ (21)

where X denotes any symmetric positive-definite matrix and (⋅)^−1/2^ denotes a combination of matrix inversion and symmetric square-rooting. On the basis of the observations recalled in the footnote ^1^, the inverse symmetric square root of a SPD matrix Γ may be computed rather inexpensively by $W\text{diag}\left(\lambda_{1}^{-1/2},\lambda_{2}^{-1/2},\ldots ,\lambda_{L}^{-1/2}\right)W^{⊤}$. Since a precision matrix $\Gamma_{j}^{\left(n\right)}$ is symmetric and positive-definite, its inverse always exists and its matrix square root always returns a symmetric, real-valued matrix. Let us remark how the introduced normalization keeps both symmetry and positive-definiteness of its argument, in fact, to what concerns symmetry:

$$\begin{array}{cc} {\left(L_{j}^{\left(n\right)}\left(X\right)\right)}^{⊤} & ={\left({\left(\Gamma_{j}^{\left(n\right)}\right)}^{-1/2}X{\left(\Gamma_{j}^{\left(n\right)}\right)}^{-1/2}\right)}^{⊤}={\left(\Gamma_{j}^{\left(n\right)}\right)}^{-⊤/2}X^{⊤}{\left(\Gamma_{j}^{\left(n\right)}\right)}^{-⊤/2}\\ & ={\left(\Gamma_{j}^{\left(n\right)}\right)}^{-1/2}X{\left(\Gamma_{j}^{\left(n\right)}\right)}^{-1/2}=L_{j}^{\left(n\right)}\left(X\right), \end{array}$$ (22)

and, to what concerns positive-definiteness:

$$\begin{array}{c} \det(L_{j}^{\left(n\right)}\left(X\right))=\det^{2}\left({\left(\Gamma_{j}^{\left(n\right)}\right)}^{-1/2}\right)\det\left(X\right)=\det\left(X\right)/\det\left(\Gamma_{j}^{\left(n\right)}\right)>0. \end{array}$$ (23)

The inverse (de-normalizing) function associated to (21) reads:

$$\begin{array}{c} {\left(L_{j}^{\left(n\right)}\right)}^{-1}\left(X\right)≔{\left(\Gamma_{j}^{\left(n\right)}\right)}^{1/2}X{\left(\Gamma_{j}^{\left(n\right)}\right)}^{1/2}. \end{array}$$ (24)

Define a precision matrix Γ normalized by (21) as $\tilde{\Gamma }≔L_{j}^{\left(n\right)}\left(\Gamma \right)$. If we apply the MEG update to ${\tilde{\Gamma }}_{j}^{\left(n\right)}$ instead of $\Gamma_{j}^{\left(n\right)}$, we get the adaptation rule

$$\begin{array}{c} {\tilde{\Gamma }}_{j}^{\left(n+1\right)}=\text{exp}(\text{log}{\tilde{\Gamma }}_{j}^{\left(n\right)}-\eta_{\text{w}}\text{sym}({}_{\frac{\partial F^{\left(n\right)}(\Gamma_{j})}{\partial {\tilde{\Gamma }}_{j}}{|}_{{\tilde{\Gamma }}_{j}={\tilde{\Gamma }}_{j}{}^{\left(n\right)}}})), \end{array}$$ (25)

where $\Gamma_{j}=\Gamma_{j}\left({\tilde{\Gamma }}_{j}\right)$ is to be thought of as a compound function, in fact, it holds that $\Gamma_{j}\left({\tilde{\Gamma }}_{j}\right)≔{\left(L_{j}^{\left(n\right)}\right)}^{-1}\left({\tilde{\Gamma }}_{j}\right)$. Notice that ${\tilde{\Gamma }}_{j}^{\left(n\right)}$ can be written as

$$\begin{array}{c} {\tilde{\Gamma }}_{j}^{\left(n\right)}=L_{j}^{\left(n\right)}\left(\Gamma_{j}^{\left(n\right)}\right)={\left(\Gamma_{j}^{\left(n\right)}\right)}^{-1/2}\Gamma_{j}^{\left(n\right)}{\left(\Gamma_{j}^{\left(n\right)}\right)}^{-1/2}=I, \end{array}$$ (26)

where $I\in R^{L\times L}$ is an identity matrix. Since log**I** = **0**, the adaptation rule (25) simplifies to

$$\begin{array}{c} {\tilde{\Gamma }}_{j}^{\left(n+1\right)}=\text{exp}(-\eta_{\text{w}}\text{sym}(\frac{\partial F^{\left(n\right)}(\Gamma_{j}({\tilde{\Gamma }}_{j}))}{\partial {\tilde{\Gamma }}_{j}}{|}_{{}_{{\tilde{\Gamma }}_{j}={\tilde{\Gamma }}_{j}{}^{\left(n\right)}}})), \end{array}$$ (27)

To find the derivative of function *F*^(*n*)^(Γ_*j*_) with respect to ${\tilde{\Gamma }}_{j}$, the following chain rule [44] is used:

$$\begin{array}{cc} {\left(\frac{\partial F^{\left(n\right)}\left(\Gamma_{j}\left({\tilde{\Gamma }}_{j}\right)\right)}{\partial {\tilde{\Gamma }}_{j}}\right)}_{kl} & =\text{Tr}[{\left(\frac{\partial F^{\left(n\right)}\left(\Gamma_{j}\right)}{\partial \Gamma_{j}}\right)}^{⊤}\frac{\partial \Gamma_{j}}{\partial {\left({\tilde{\Gamma }}_{j}\right)}_{kl}}]\\ & =\text{Tr}[{\left(\frac{\partial F^{\left(n\right)}\left(\Gamma_{j}\right)}{\partial \Gamma_{j}}\right)}^{⊤}{\left(\Gamma_{j}^{\left(n\right)}\right)}^{1/2}\frac{\partial {\tilde{\Gamma }}_{j}}{\partial {\left({\tilde{\Gamma }}_{j}\right)}_{kl}}{\left(\Gamma_{j}^{\left(n\right)}\right)}^{1/2}]\\ & =\text{Tr}[{\left(\frac{\partial F^{\left(n\right)}\left(\Gamma_{j}\right)}{\partial \Gamma_{j}}\right)}^{⊤}{\left(\Gamma_{j}^{\left(n\right)}\right)}^{1/2}S_{kl}{\left(\Gamma_{j}^{\left(n\right)}\right)}^{1/2}]\\ & =({\left(\Gamma_{j}^{\left(n\right)}\right)}^{1/2}{\left(\frac{\partial F^{\left(n\right)}(\Gamma_{j})}{\partial \Gamma_{j}}\right)}^{⊤}(\Gamma_{j}^{\left(n\right)}){{}^{1/2})}_{lk}, \end{array}$$ (28)

where the notation (**X**)_*kl*_ indicates the (*k*, *l*)-th entry of matrix **X**, Tr(·) denotes matrix trace, and **S**_*kl*_ is the single-entry matrix [44], whose (*k*, *l*)-th entry is 1 and each other entry takes the value 0. From the property (28), we get

$$\begin{array}{cc} \frac{\partial F^{\left(n\right)}\left(\Gamma_{j}\left({\tilde{\Gamma }}_{j}\right)\right)}{\partial {\tilde{\Gamma }}_{j}} & ={\left({\left(\Gamma_{j}^{\left(n\right)}\right)}^{1/2}{\left(\frac{\partial F^{\left(n\right)}\left(\Gamma_{j}\right)}{\partial \Gamma_{j}}\right)}^{⊤}{\left(\Gamma_{j}^{\left(n\right)}\right)}^{1/2}\right)}^{⊤}\\ & ={\left(\Gamma_{j}^{\left(n\right)}\right)}^{1/2}\frac{\partial F^{\left(n\right)}\left(\Gamma_{j}\right)}{\partial \Gamma_{j}}{\left(\Gamma_{j}^{\left(n\right)}\right)}^{1/2}, \end{array}$$ (29)

thanks to the symmetry of the involved matrices and expressions. Using the formula (29), the adaptation rule (27) can be written as

$$\begin{array}{cc} {\tilde{\Gamma }}_{j}^{\left(n+1\right)} & =\exp(-\eta_{w}\;\text{sym}({\left(L_{j}^{\left(n\right)}\right)}^{-1}({\frac{\partial F^{\left(n\right)}\left(\Gamma_{j}\right)}{\partial \Gamma_{j}}|}_{\Gamma_{j}={\Gamma_{j}}^{\left(n\right)}})))\\ & =\exp(-\eta_{w}\;{\left(L_{j}^{\left(n\right)}\right)}^{-1}(\text{sym}({\frac{\partial F^{\left(n\right)}\left(\Gamma_{j}\right)}{\partial \Gamma_{j}}|}_{\Gamma_{j}={\Gamma_{j}}^{\left(n\right)}}))). \end{array}$$ (30)

Thanks to the normalizing function, we can update the precision matrices stably. Then, the (*n* + 1)-th precision matrix is obtained by applying the inverse normalizing function. Therefore, the following update rule is derived:

$$\begin{array}{cc} \Gamma_{j}^{\left(n+1\right)} & ={\left(L_{j}^{\left(n\right)}\right)}^{-1}\left({\tilde{\Gamma }}_{j}^{\left(n+1\right)}\right)={\left(\Gamma_{j}^{\left(n\right)}\right)}^{1/2}{\tilde{\Gamma }}_{j}^{\left(n+1\right)}{\left(\Gamma_{j}^{\left(n\right)}\right)}^{1/2}\\ & ={\left(\Gamma_{j}^{\left(n\right)}\right)}^{1/2}\exp(-\eta_{w}\;{\left(\Gamma_{j}^{\left(n\right)}\right)}^{1/2}\text{sym}({\frac{\partial F^{\left(n\right)}\left(\Gamma_{j}\right)}{\partial \Gamma_{j}}|}_{\Gamma_{j}={\Gamma_{j}}^{\left(n\right)}}){\left(\Gamma_{j}^{\left(n\right)}\right)}^{1/2}){\left(\Gamma_{j}^{\left(n\right)}\right)}^{1/2}. \end{array}$$ (31)

From (31), we can see that unlike (20), this adaptation rule dose not require the computation of logΓ. We call this adaptation rule normalized matrix exponentiated gradient (NMEG).

As a special instance, let us consider the case *L* = 1. The NMEG update rule in the case of *L* = 1 can be derived by replacing each precision matrix Γ with a scalar parameter *γ* > 0 in (31):

$$\begin{array}{c} \gamma_{j}^{\left(n+1\right)}=\gamma_{j}^{\left(n\right)}\exp(-\eta_{w}\gamma_{j}^{\left(n\right)}{\frac{\partial F^{\left(n\right)}\left(\gamma_{j}\right)}{\partial \gamma_{j}}|}_{\gamma_{j}=\gamma_{j}^{\left(n\right)}}), \end{array}$$ (32)

which apparently keeps each parameter *γ*_*j*_ in the positive half-line during adaptation. The partial derivative of the cost function, in this case, reads

$$\begin{array}{c} {\frac{\partial F^{\left(n\right)}\left(\gamma_{j}\right)}{\partial \gamma_{j}}|}_{\gamma_{j}=\gamma_{j}^{\left(n\right)}}=-2\gamma_{j}^{\left(n\right)}e^{\left(n\right)}h_{j}^{\left(n\right)}\kappa \left(u^{\left(n\right)},c_{j}^{\left(n\right)};\gamma_{j}^{\left(n\right)}\right){∥u^{\left(n\right)}-c_{j}^{\left(n\right)}∥}^{2}. \end{array}$$ (33)

Such special case was proposed and discussed in the contributions [6, 28].

The adaptation rule (31) was derived on the basis of matrix normalization, therefore, it is legitimate to wonder if it constitutes a valid algorithm to update a matrix in the space of SPD tensors. The answer is positive, indeed, since the rule (31) may be regarded as an application of a general geodesic-based stepping rule on the manifold of symmetric positive-definite matrices endowed with the canonical metric, namely

$$\begin{array}{cc} & \Gamma_{j}^{\left(n+1\right)}=g_{\Gamma_{j}^{\left(n\right)}}(-\eta_{w}\;\Gamma_{j}^{\left(n\right)}\text{sym}({\frac{\partial F^{\left(n\right)}\left(\Gamma_{j}\right)}{\partial \Gamma_{j}}|}_{\Gamma_{j}={\Gamma_{j}}^{\left(n\right)}})\Gamma_{j}^{\left(n\right)}), \end{array}$$ (34)

where the function *g*_*X*_(*V*) denotes a geodesic arc in the SPD space departing from a point *X* in the direction *V* and is given by

$$\begin{array}{c} g_{X}\left(V\right)≔X^{1/2}\exp\left(X^{-1/2}VX^{-1/2}\right)X^{1/2}, \end{array}$$ (35)

as explained, for example, in [43] and [45]. Notice, in addition, that the argument of the function *g* in (34) is proportional to the Riemannian gradient of the criterion function *F* with respect to the canonical metric, as defined in the previous Subsection 3.3.1.

### 3.4 Sparse KNLMS incorporated with generalized Gaussian kernel parameters

To avoid overfitting and to prevent monotonic growth of a dictionary, the proposed adaptation rules for the generalized Gaussian parameters are applied jointly with an ℓ_1_-regularization [33]. The proposed method is summarized in Algorithm 1.

**Algorithm 1** Dictionary Learning for Generalized Gaussian Kernel Adaptive Filtering

1: Set precision matrices of kernels Γ_init_.

2: Set the initial center vector ***c***^(0)^ ← ***u***^(0)^

3: Add (***c***^(0)^, Γ_init_) into the dictionary as the 1st member, $D^{\left(0\right)}\leftarrow \left\{\left(c^{\left(0\right)},\Gamma_{\text{init}}\right)\right\}$.

4: **for** *n* > 1 **do**

5:  Set the *n*-th center vector **c**^(*n*)^ ← ***u***^(*n*)^

6:  Add (***c***^(*n*)^, Γ_init_) to the dictionary as a new member, $D^{\left(n\right)}\leftarrow D^{\left(n-1\right)}\cup \left\{\left(c^{\left(n\right)},\Gamma_{\text{init}}\right)\right\}$.

7:  **for** *j* ← 0 to size of $D^{\left(n\right)}-1$ **do**

8:   Update the center vectors $c_{j}^{\left(n\right)}$ using (18).

9:   Update the precision matrices $\Gamma_{j}^{\left(n\right)}$ using MEG (20) or NMEG (31).

10:   Update the filter coefficients *h*_*j*_ according to a forward-backward splitting scheme (8).

11:  **end for**

12:  **for** *j* such that *h*_*j*_ = 0 **do**

13:    Remove the *j*-th element from the dictionary $D^{\left(n\right)}$.

14:  **end for**

15:  *n* ← *n* + 1

16: **end for**

## 4 Numerical experiments

In this section, we compare the KNLMS-ℓ_1_ [33], the NMEG (*L* = 1) [6, 28] in (32), the MEG in (20), and the NMEG in (31) through three types of simulations. The first simulation is a time series prediction in a toy model defined by Gaussian functions with scalar widths. The second simulation is an online prediction in a toy model defined by Gaussian functions with precision matrices. The last simulation consists in an online prediction of the state of a Lorenz chaotic system. In these simulations, mean squared error (MSE) and mean dictionary size were adopted as the evaluation criteria. Both indices were averaged over 200 independent trials to compensate for statistical fluctuations in each single trial.

### 4.1 Time series prediction in a toy signal model constructed by standard Gaussian functions

Consider the following synthetic signal model:

$$\begin{array}{c} d^{\left(n\right)}≔10\exp(-5∥u^{\left(n\right)}-{\left[3,3\right]}^{⊤}{∥}^{2})+10\exp(-0.2∥u^{\left(n\right)}-{\left[7,7\right]}^{⊤}{∥}^{2}), \end{array}$$ (36)

corrupted by an additive zero-mean white Gaussian noise with standard deviation equal to 0.3. The input samples ***u***^(*n*)^ are drawn from a 2-dimensional uniform distribution with support [0, 10] × [0, 10]. The parameters values for the learning schemes utilized in this experiment are given in Table 2. In addition, the parameters values for the forward-backward splitting scheme are λ = 0.09 and *σ* = 0.03.

**Table 2:** Values of learning parameters in the experiment described in Subsection 4.1.

Learning algorithm	Parameters values
KNLMS-ℓ_1_	*γ* = 1.0, *μ* = 1.0 × 10^−3^, *ρ* = 0.1
NMEG (*L* = 1)	*γ*_init._ = 1.0, *μ* = 1.0 × 10^−3^ *ρ* = 0.1, *η_c_* = 1.0 × 10^−3^, *η*_w_ = 0.05
MEG	Γ_init_ = ***I***, *μ* = 1.0 × 10^−3^ *ρ* = 0.1, *η_c_* = 1.0 × 10^−3^, *η*_w_ = 0.05, *L* = 2
NMEG	Γ_init_ = ***I***, *μ* = 1.0 × 10^−3^ *ρ* = 0.1, *η_c_* = 1.0 × 10^−3^, *η*_w_ = 0.05, *L* = 2

**Fig 3:**
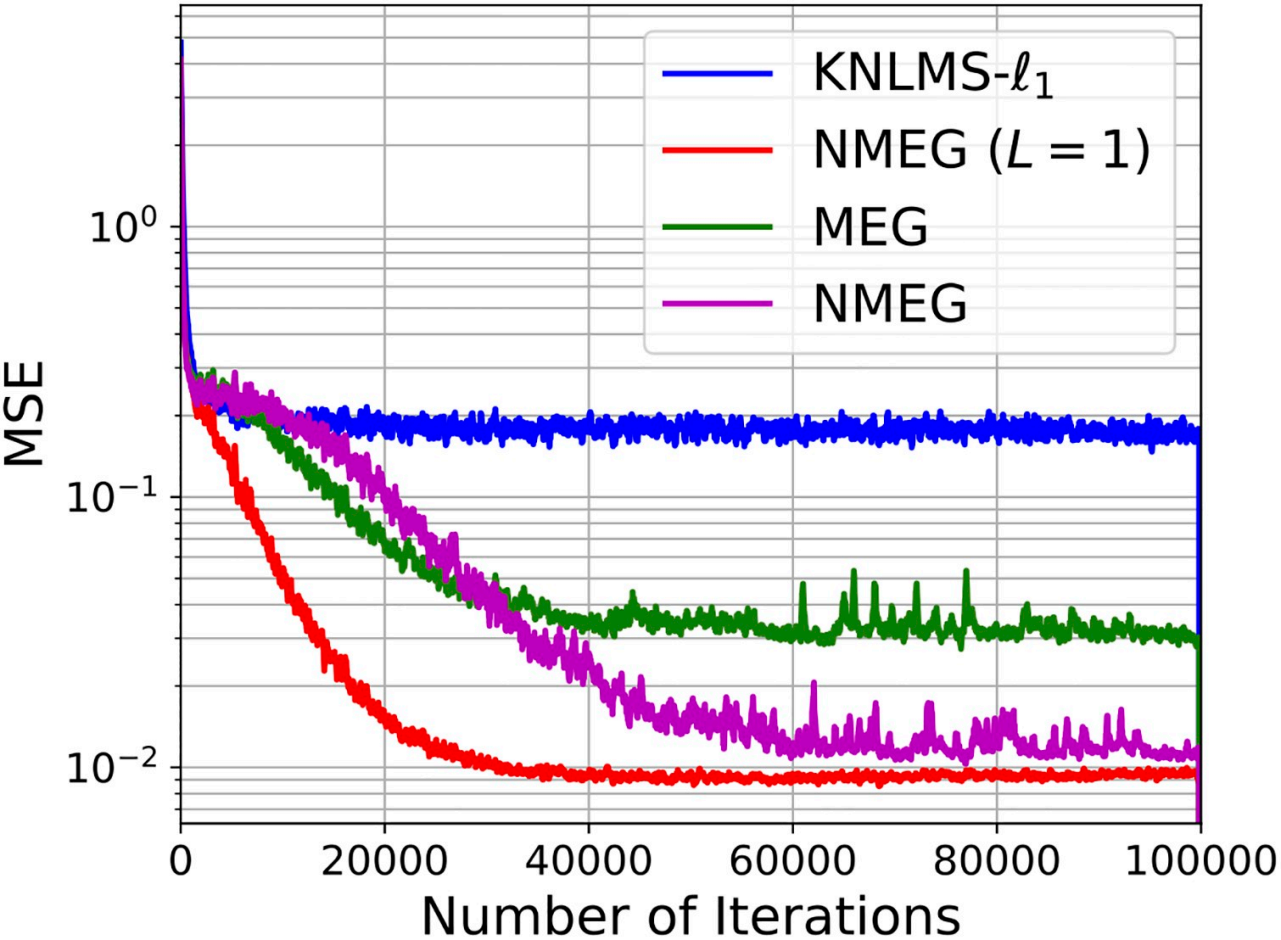
Convergence curves of filters in the experiment described in the Section 4.1. These results were obtained as averages over 200 independent trials.

**Fig 4:**
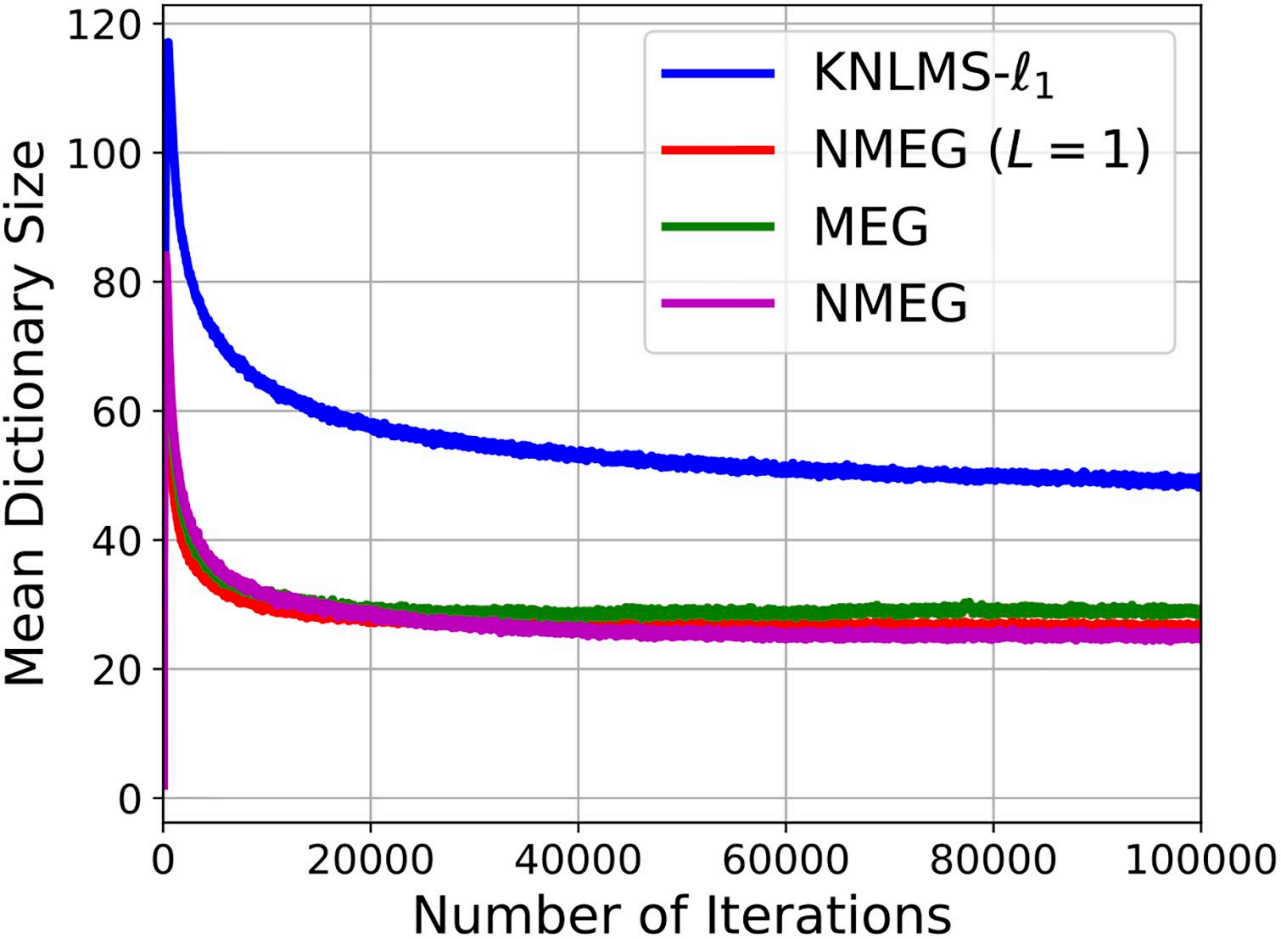
Dictionary size evolution in experiment described in the Section 4.2. These results were obtained as averages over 200 independent trials.

Figs 3 and 4 show the mean squared error and mean dictionary size of filters at each iteration, respectively. In Fig 3, the NMEG (*L* = 1), the MEG, and the NMEG show lower MSE than the KNLMS-ℓ_1_. This confirms the efficacy of updating the scalar widths *γ* and the precision matrices Γ. The NMEG (*L* = 1) converges faster than the other algorithms in this comparison. However, when the iteration index *n* reaches about 100, 000, the NMEG (*L* = 1) and NMEG exhibit almost the same mean MSE even though the NMEG uses generalized Gaussian kernels. The Fig 4 shows that the NMEG (*L* = 1), the MEG, and the NMEG are able to keep a small dictionary size.

### 4.2 Time series prediction in a toy signal model constructed by generalized Gaussian functions

Further, consider the following synthetic signal model:

$$\begin{array}{cc} d^{\left(n\right)}≔ & 10\exp(-{\left(u^{\left(n\right)}-{\left[3,3\right]}^{⊤}\right)}^{⊤}\Lambda \left(u^{\left(n\right)}-{\left[3,3\right]}^{⊤}\right))+\\ & 10\exp(-{\left(u^{\left(n\right)}-{\left[7,7\right]}^{⊤}\right)}^{⊤}\Lambda \left(u^{\left(n\right)}-{\left[7,7\right]}^{⊤}\right)), \end{array}$$ (37)

corrupted by the same kind of noise, and driven by the same input sequence, as in the previous experiment. Parameters values pertaining to learning schemes utilized in this experiment are given in Table 3. In addition, the parameters values for the forward-backward splitting scheme are λ = 0.09 and *σ* = 0.03.

**Table 3:** Values of learning parameters in the experiment described in Subection 4.2.

Learning algorithm	Parameters values
KNLMS-ℓ_1_	*ρ* = 0.03, *γ* = 1.0, *μ* = 1.0 × 10^−3^, *β* = 0.1
NMEG (*L* = 1)	*ρ* = 0.03, *γ*_init_ = 1.0, *μ* = 1.0 × 10 ^−3^ *β* = 0.1, *η_c_* = 1.0 × 10^−3^, *η_w_* = 0.05
MEG	*ρ* = 0.03, Γ_init_ = ***I***, *μ* = 1.0 × 10 ^−3^ *β* = 0.1, *η_c_* = 1.0 × 10^−3^, *η_w_* = 0.05, *L* = 2
NMEG	*ρ* = 0.03, Γ_init_ = ***I***, *μ* = 1.0 × 10 ^−3^ *β* = 0.1, *η_c_* = 1.0 × 10^−3^, *η_w_* = 0.05, *L* = 2

**Fig 5:**
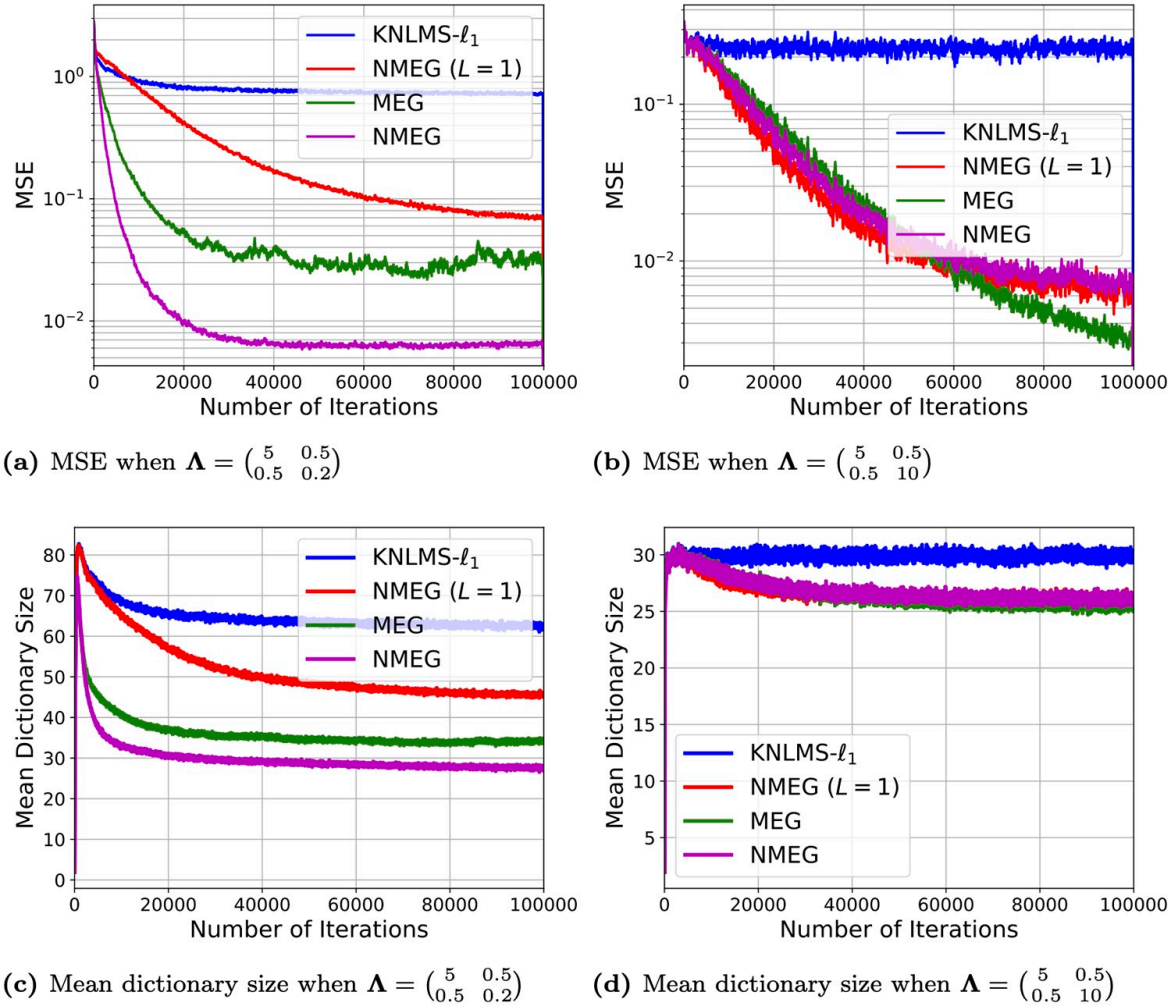
Performance comparison in experiment described in the Section 4.1. The learning curves of MSE ((a) and (b)) and mean dictionary size ((c) and (d)) for two different matrices **Λ**. These results were obtained as averages over 200 independent trials.

We tested the behavior of the proposed adaptive kernel filter theory on two different cases characterized by two instances of **Λ**:

$$\begin{array}{c} (\begin{array}{cc} 5 & 0.5\\ 0.5 & 0.2 \end{array}),(\begin{array}{cc} 5 & 0.5\\ 0.5 & 10 \end{array}), \end{array}$$ (38)

which have, as smallest eigenvalues, 0.148 and 4.95, respectively. The Fig 5 shows the mean MSE and mean dictionary size of filters at each iteration. In Fig 5a and 5b, the MEG and the NMEG show lower MSE than the KNLMS-ℓ_1_ and NMEG (*L* = 1).

The obtained results confirm the efficacy of using (adaptive) generalized Gaussian kernels. Comparing the MSE curves of the MEG and of the NMEG, it is immediate to see how the performance of the MEG algorithm degrades when the matrix **Λ** is close to singularity, namely when $\Lambda =(\begin{array}{cc} 5 & 0.5\\ 0.5 & 0.2 \end{array})$, which implies that the term $\log\Gamma_{j}^{\left(n\right)}$ in (20) is difficult to compute, while the NMEG is able to perform well in both cases. The Fig 5c and 5d confirm that the NMEG produces the smallest dictionary. The above results clearly confirm the efficacy of the proposed normalization method for updating precision matrices.

### 4.3 Modeling of a Lorenz chaotic system

**Fig 6:**
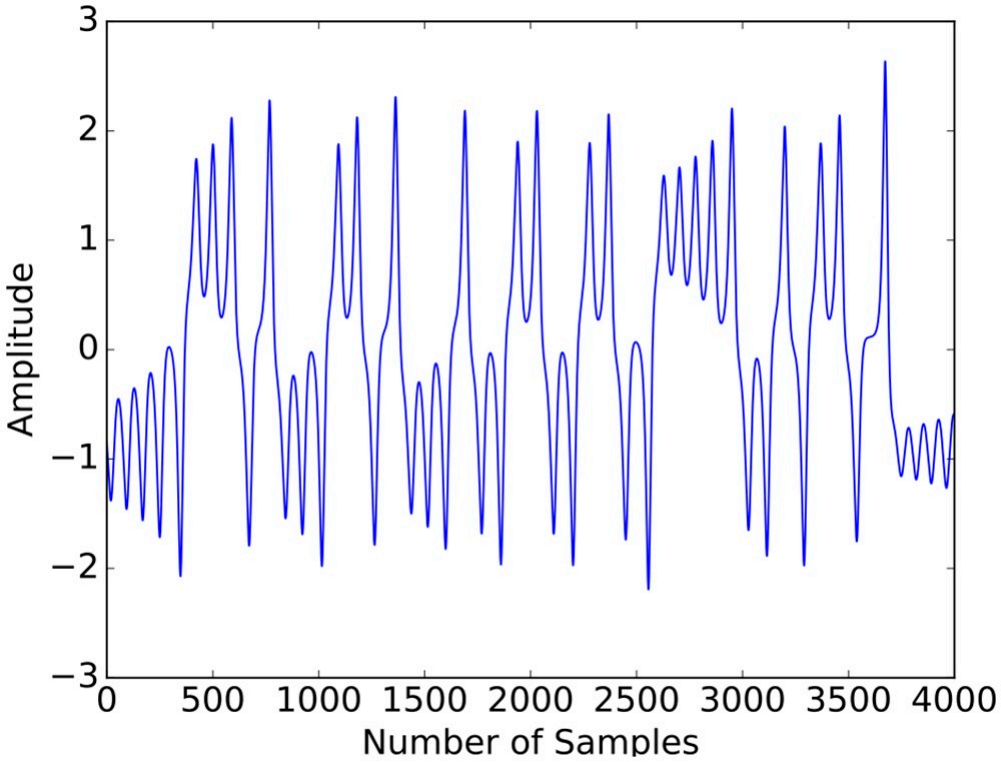
Segment of the processed Lorenz time series (*x*-component of the flow of the system (39)).

Adaptive kernel filters are widely used in time-series prediction [46]. We tested the devised algorithm to model a Lorentz chaotic system [30]:

$$\begin{array}{c} \left\{ \begin{array}{cc} \frac{dx}{dt}= & -\alpha x+yz\\ \frac{dy}{dt}= & -\delta (y-z)\\ \frac{dz}{dt}= & -xy+\theta y-z, \end{array}\right. \end{array}$$ (39)

where *α* = 8/3, *δ* = 10, and *θ* = 28 [11]. The continuous-time equations were sampled by subdividing each unitary interval in 100 sub-intervals. The *x* component was used to test the algorithm’s prediction ability. The *x* time series was normalized to zero-mean and unit variance. A segment of such time series is displayed in Fig 6.

The input signal to the modeling algorithm was constructed as ***u***^(*n*)^ = [*x*^(*n*−5)^, *x*^(*n*−4)^, …, *x*^(*n*−1)^]^⊤^ and the current value *x*^(*n*)^ was taken as the desired response. The values of the learning parameters in this experiment are given in the Table 4. In addition, the parameters for the forward-backward splitting scheme are λ = 0.5 and *σ* = 0.05.

**Table 4:** Values of the parameters in the experiment explained in Section 4.3.

Learning algorithm	Parameters values
KNLMS-ℓ_1_	*μ* = 0.5, *ρ* = 0.05, *γ* = 1.0, *β* = 0.1
NMEG (*L* = 1)	*μ* = 0.5, *ρ* = 0.05, *γ*_init_ = 1.0 *β* = 0.1, *η_c_* = 0.5, *η_w_* = 0.1
MEG	*μ* = 0.5, *ρ* = 0.05, Γ_init_ = ***I*** *β* = 0.1, *η_c_* = 0.5, *η_w_* = 0.1, *L* = 5
NMEG	*μ* = 0.5, *ρ* = 0.05, Γ_init_ = ***I*** *β* = 0.1, *η_c_* = 0.5, *η_w_* = 0.1, *L* = 5

**Fig 7:**
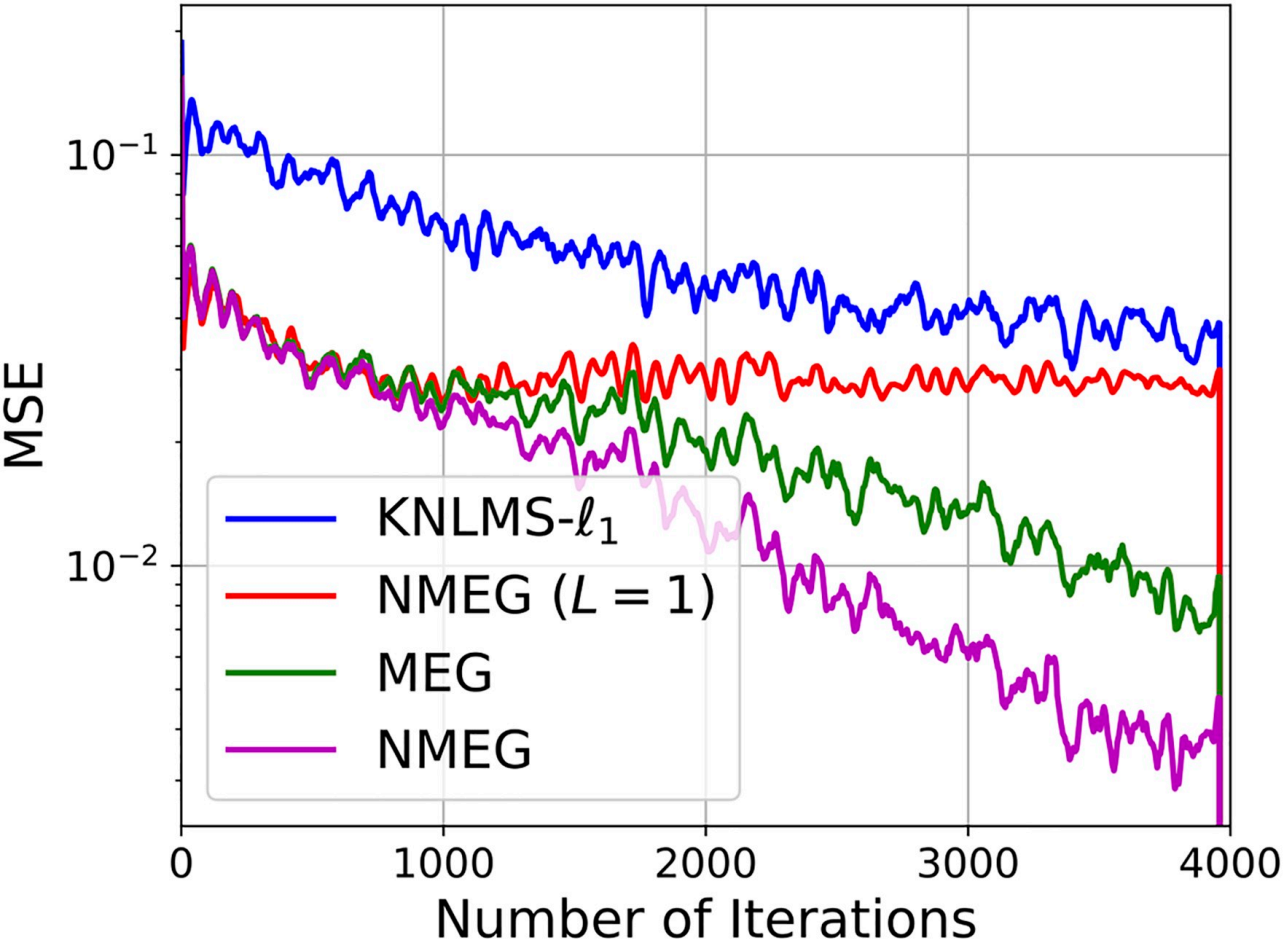
Convergence curves in the experiment described in the Section 4.3. These results were obtained as the average over 200 independent trials with different segments of the Lorenz time series.

**Fig 8:**
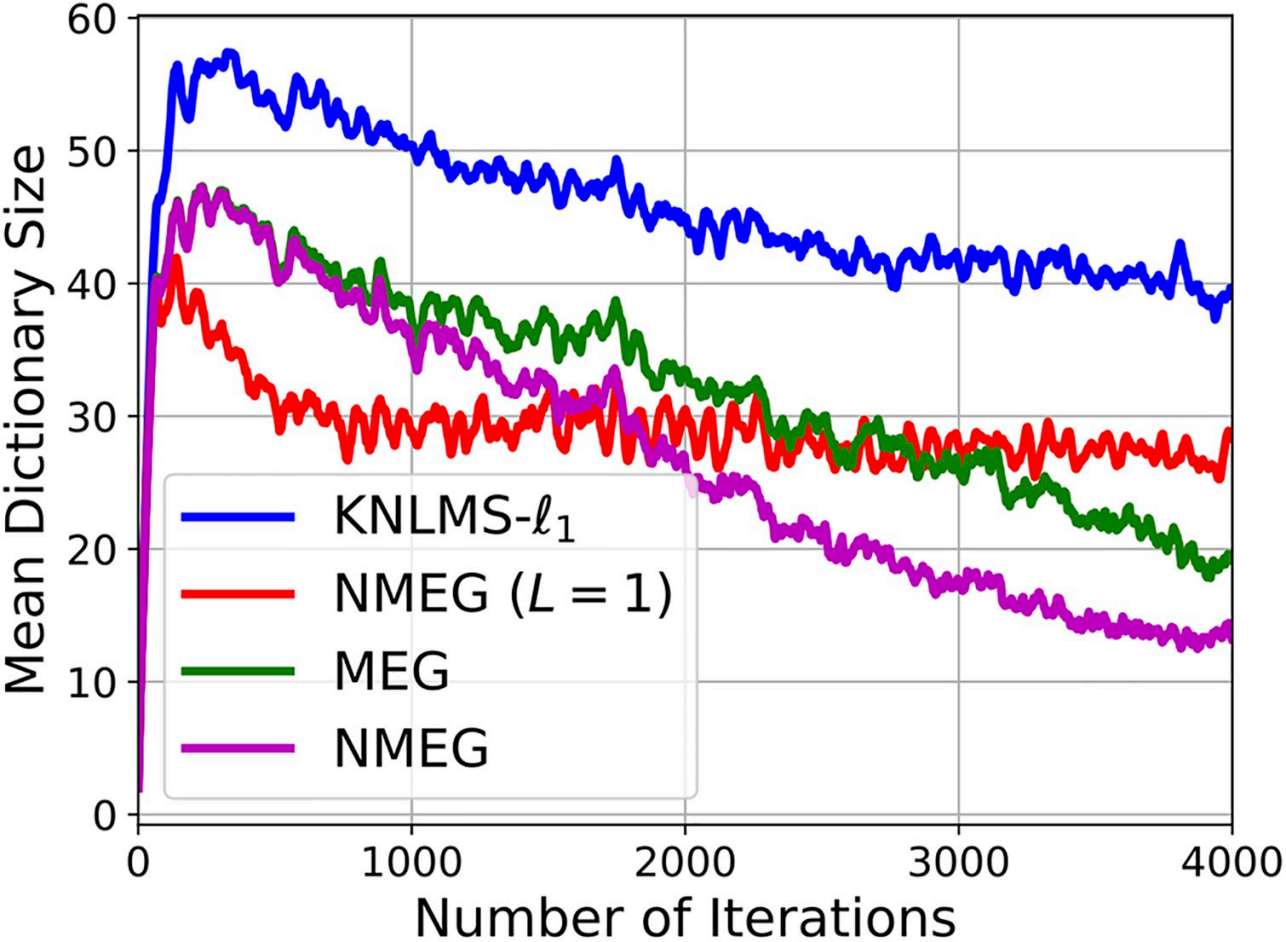
Dictionary size evolution in the experiment described in the Section 4.3. These results were obtained as the average over 200 independent trials with different segments of the Lorenz time series.

The Figs 7 and 8 show the MSE and the mean dictionary size at each iteration, respectively.

**Fig 9:**
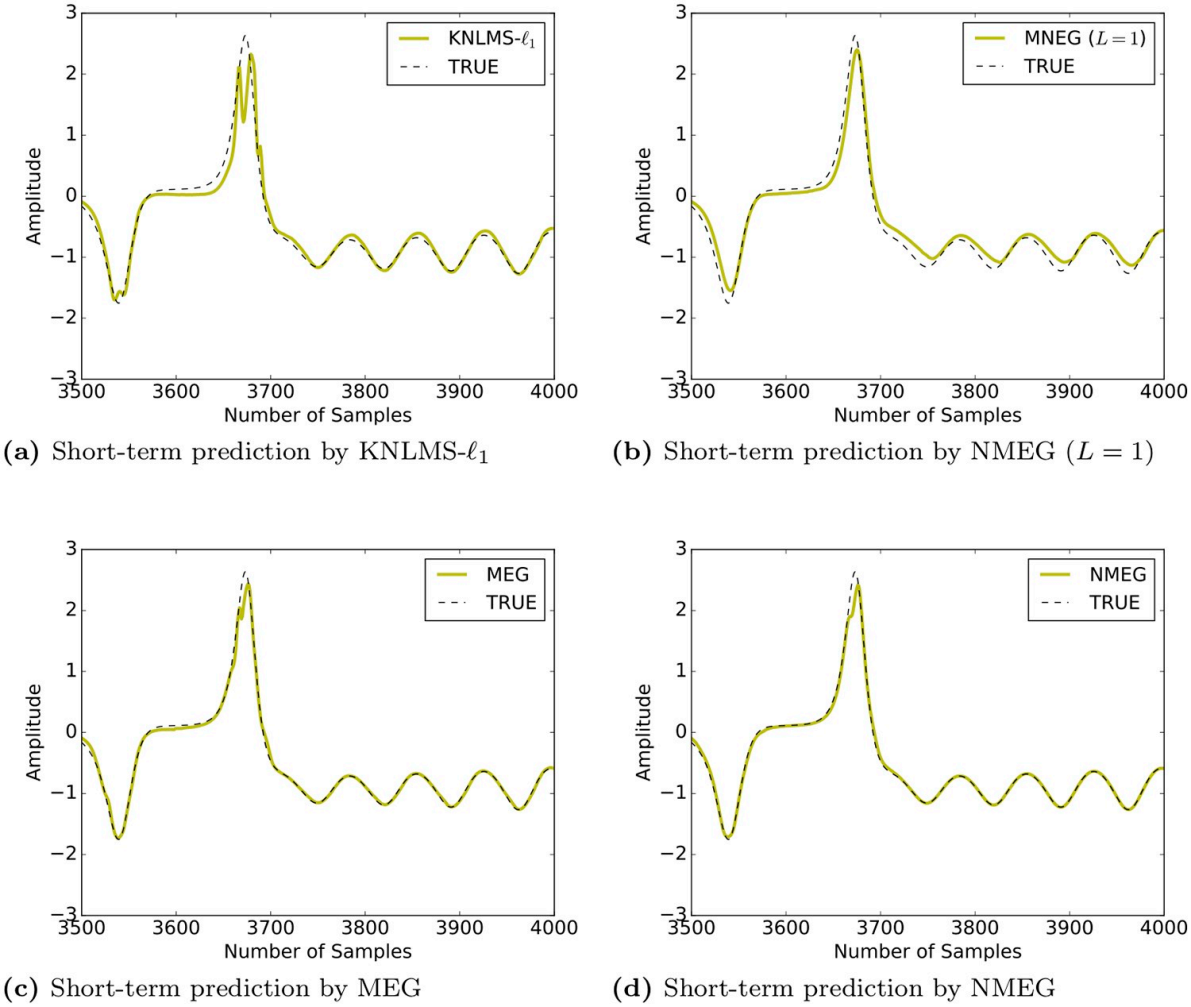
Prediction of the state of a Lorenz chaotic system. (Plots of the last 500 samples).

Simulation results indicate that the proposed MEG and NMEG exhibit much better performances, namely, they achieve much smaller mean dictionary size and much smaller MSE values than the other algorithms used for comparison. Comparing the MEG algorithm with the NMEG, the NMEG exhibits better performance in terms of both MSE and mean dictionary size although their parameters are set to the same values. The Fig 9 shows a result of short-term prediction of the Lorenz time series. It can be seen that the NMEG has higher tracking ability than the NMEG (*L* = 1). This result confirms the validity of the proposed model in the case that the components of the input signals are mutually correlated.

## 5 Conclusions

This paper proposed a flexible dictionary learning strategy in the context of generalized Gaussian kernel adaptive filtering, where the kernel parameters are all adaptive and data driven. We introduced a novel update rule for precision matrices, which allows one to update each precision matrix stably thanks to an effective normalization. The main advantage of the proposed approach is that the number of parameters in the proposed generalized Gaussian kernels is larger than the number of parameters in the conventional kernel functions. The adaptation rule of kernel parameters are successfully established within a Lie-group theoretic setting. In addition, together with the ℓ_1_ regularized least squares, the overall kernel adaptive filtering algorithms can avoid overfitting and monotonic inflation of a dictionary. Numerical tests confirmed that the proposed algorithm entails lesser mean squared error and dictionary size in modeling nonlinear systems.

## References

[1] Haykin S. Adaptive Filter Theory. Upper Saddle River, NJ: Prentice-Hall; 2002.

[2] Aronszajn N. Theory of reproducing kernels. Trans Amer Math Soc. 1950;68(9):337–404.

[3] Garcia-Vega S, Zeng XJ, Keane J. Stock price prediction using kernel adaptive filtering within a stock market interdependence approach. Social Science Research Network. 2018.

[4] Van Vaerenbergh S, Azpicueta-Ruiz LA, Comminiello D. A split kernel adaptive filtering architecture for nonlinear acoustic echo cancellation. In: 2016 24th European Signal Processing Conference (EUSIPCO); 2016. p. 1768–1772.

[5] Fawad, Jamil Khan M, Rahman MU, Amin Y, Tenhunen H. Low-rank multi-channel features for robust visual object tracking. Symmetry. 2019;11(9). 10.3390/sym11091155

[6] Wada T, Fukumori K, Tanaka T. Dictionary learning for Gaussian kernel adaptive filtering with variable kernel center and width. In: Proc. of 2018 IEEE International Conference on Acoustics, Speech and Signal Processing (ICASSP 2018); 2018. p. 2766–2770.

[7] Kivinen J, Smola AJ, Williamson RC. Online learning with kernels. IEEE Trans Signal Process. 2004;52(8):2165–2176. 10.1109/TSP.2004.830991

[8] Liu W, Principe J, Haykin S. Kernel Adaptive Filtering. Hoboken, NJ: Wiley; 2010.

[9] Liu W, Pokharel PP, Principe JC. The kernel least-mean-square algorithm. IEEE Trans Signal Process. 2008;56(2):543–554. 10.1109/TSP.2007.907881

[10] Bouboulis P, Theodoridis S. Extension of Wirtinger’s calculus to reproducing kernel Hilbert spaces and the complex kernel LMS. IEEE Trans Signal Process. 2011;59(3):964–978. 10.1109/TSP.2010.2096420

[11] Chen B, Zhao S, Zhu P, Principe JC. Quantized kernel least mean square algorithm. IEEE Trans Neural Netw Learn Syst. 2012;23(1):22–32. 10.1109/TNNLS.2011.2178446 24808453

[12] Tobar FA, Kung SY, Mandic DP. Multikernel least mean square algorithm. IEEE Trans Neural Netw. 2014;25(2):265–277. 10.1109/TNNLS.2013.2272594 24807027

[13] Liu W, Principe JC. Kernel affine projection algorithms. EURASIP J Adv Signal Process. 2008;2008(1):1–13.

[14] Gil-Cacho JM, van Waterschoot T, Moonen M, Jensen SH. Nonlinear acoustic echo cancellation based on a parallel-cascade kernel affine projection algorithm. In: Proc. of 2012 IEEE International Conference on Acoustics, Speech, and Signal Processing (ICASSP 2012); 2012. p. 33–36.

[15] Engel Y, Mannor S, Meir R. The kernel recursive least-squares algorithm. IEEE Trans Signal Process. 2004;52(8):2275–2285. 10.1109/TSP.2004.830985

[16] Raja MAZ, Chaudhary NI, Ahmed Z, Rehman AU, Aslam MS. A novel application of kernel adaptive filtering algorithms for attenuation of noise interferences. Neural Comput Appl. 2019;31(12):9221–9240. 10.1007/s00521-019-04390-8

[17] Chaudhary NI, Zubair S, Aslam MS, Raja MAZ, Tenreiro Machado JA. Design of momentum fractional LMS for Hammerstein nonlinear system identification with application to electrically stimulated muscle model. The European Physical Journal Plus. 2019;134(8). 10.1140/epjp/i2019-12785-8

[18] Raja MAZ, Akhtar R, Chaudhary NI, Zhiyu Z, Khan Q, Rehman AU, et al A new computing paradigm for the optimization of parameters in adaptive beamforming using fractional processing. The European Physical Journal Plus. 2019;134(6). 10.1140/epjp/i2019-12654-6

[19] Chaudhary NI, Aslam KZ, Zubair S, Raja MAZ, Dedovic N. Normalized fractional adaptive methods for nonlinear control autoregressive systems. Appl Math Model. 2019;66:457–471. 10.1016/j.apm.2018.09.028

[20] Aslam MS, Raja MAZ. A new adaptive strategy to improve online secondary path modeling in active noise control systems using fractional signal processing approach. Signal Process. 2015;107(7):433–443.

[21] Saide C, Lengelle R, Honeine P, Richard C, Achkar R. Dictionary adaptation for online prediction of time series data with kernels. In: Proc. of 2012 IEEE Statistical Signal Processing Workshop (SSP); 2012. p. 604–607.

[22] Saide C, Lengelle R, Honeine P, Achkar R. Online kernel adaptive algorithms with dictionary adaptation for MIMO models. IEEE Signal Process Lett. 2013;20(5):535–538. 10.1109/LSP.2013.2254711

[23] Ishida T, Tanaka T. Efficient construction of dictionaries for kernel adaptive filtering in a dynamic environment. In: Proc. of 2015 IEEE International Conference on Acoustics, Speech and Signal Processing (ICASSP 2015); 2015. p. 3536–3540.

[24] Benoudjit N, Verleysen M. On the kernel widths in radial-basis function networks. Neural Process Lett. 2003;18(2):139–154. 10.1023/A:1026289910256

[25] Ghosh AK. Kernel discriminant analysis using case-specific smoothing parameters. IEEE Trans Syst, Man, Cybern B. 2008;38(5):1413–1418. 10.1109/TSMCB.2008.925754 18784021

[26] Chen B, Liang J, Zheng N, Principe JC. Kernel least mean square with adaptive kernel size. Neurocomputing. 2016;191:95–106. 10.1016/j.neucom.2016.01.004

[27] Fan H, Song Q, Shrestha SB. Kernel online learning with adaptive kernel width. Neurocomputing. 2016;175:233–242. 10.1016/j.neucom.2015.10.055

[28] Wada T, Tanaka T. Doubly adaptive kernel filtering. In: Proc. of 2017 Asia-Pacific Signal and Information Processing Association Annual Summit and Conference (APSIPA 2017). TA-P3.6; 2017. p. 904–909.

[29] Platt J. A resource-allocating network for function interpolation. Neural computation. 1991;3(2):213–225. 10.1162/neco.1991.3.2.213 31167310

[30] Liu W, Park I, Wang Y, Principe JC. Extended kernel recursive least squares algorithm. IEEE Trans Signal Process. 2009;57(10):3801–3814. 10.1109/TSP.2009.2022007

[31] Richard C, Bermudez JCM, Honeine P. Online prediction of time series data with kernels. IEEE Trans Signal Process. 2009;57(3):1058–1067. 10.1109/TSP.2008.2009895

[32] Gao W, Chen J, Richard C, Huang J, Flamary R. Kernel LMS algorithm with forward-backward splitting for dictionary learning. In: Proc. of 2013 IEEE International Conference on Acoustics, Speech and Signal Processing (ICASSP 2013); 2013. p. 5735–5739.

[33] Gao W, Chen J, Richard C, Huang J. Online dictionary learning for kernel LMS. IEEE Trans Signal Process. 2014;62(11):2765–2777. 10.1109/TSP.2014.2318132

[34] Wang YXR, Liu K, Theusch E, Rotter JI, Medina MW, Waterman MS, et al Generalized correlation measure using count statistics for gene expression data with ordered samples. Bioinformatics. 2017;34(4):617–624. 10.1093/bioinformatics/btx641 PMC5860612 29040382

[35] Harasym J, Olędzki R. The mutual correlation of glucose, starch, and beta-glucan release during microwave heating and antioxidant activity of oat water extracts. Food Bioproc Tech. 2018;11(4):874–884. 10.1007/s11947-018-2065-x

[36] Balli T, Palaniappan R. Nonlinear Approach to Brain Signal Modeling. In: Encyclopedia of Information Science and Technology. IGI Global—Publisher of Timely Knowledge; 2009.

[37] Murakami Y, Yamagishi M, Yukawa M, Yamada I. A sparse adaptive filtering using time-varying soft-thresholding techniques. In: Proc. of 2010 IEEE International Conference on Acoustics, Speech and Signal Processing (ICASSP 2010); 2010. p. 3734–3737.

[38] Ishida T, Tanaka T. Multikernel adaptive filters with multiple dictionaries and regularization. In: Proc. of 2013 Asia-Pacific Signal and Information Processing Association Annual Summit and Conference (APSIPA 2013); 2013. p. 1–6.

[39] Wada T, Tanaka T. Dictionary adaptation for adaptive filtering with multiple kernels in a dynamic environment. In: Proc. of 2016 IEEE International Conference on Signal Processing, Communications and Computing (ICSPCC 2016). IEEE; 2016. p. 1–4.

[40] Yukawa M. Multikernel adaptive filtering. IEEE Trans Signal Process. 2012;60(9):4672–4682. 10.1109/TSP.2012.2200889

[41] Tsuda K, Rätsch G, Warmuth MK. Matrix exponentiated gradient updates for on-line learning and Bregman projection. J Mach Learn Res. 2005;6(Jun):995–1018.

[42] Fiori S. Lie-group-type neural system learning by manifold retractions. Neural Networks. 2008;21(10):1524–1529. 10.1016/j.neunet.2008.09.009 18980831

[43] Fiori S. Learning the Fréchet mean over the manifold of symmetric positive-definite matrices. Cogn Comp. 2009;1(4):279–291. 10.1007/s12559-009-9026-7

[44] Petersen KB, Pedersen MS. The Matrix Cookbook. Version: November 15, 2012; 2012. https://www.math.uwaterloo.ca/~hwolkowi/matrixcookbook.pdf.

[45] Uehara T, Sartori M, Tanaka T, Fiori S. Robust averaging of covariances for EEG recordings classification in motor imagery brain computer interfaces. Neural Comput. 2017;29(6):1631–1666. 10.1162/NECO_a_00963 28410052

[46] Xue N, Luo X, Gao Y, Wang W, Wang L, Huang C, et al Kernel mixture correntropy conjugate gradient algorithm for time series prediction. Entropy. 2019;21(8). 10.3390/e21080785 PMC7515314 33267498

